# Fourth-order fitted mesh scheme for semilinear singularly perturbed reaction–diffusion problems

**DOI:** 10.1186/s13104-023-06631-5

**Published:** 2023-11-29

**Authors:** Birtukan Tebabal Reda, Tesfaye Aga Bullo, Gemechis File Duressa

**Affiliations:** Department of Mathematics, College of Natural Science, Jimma University, Jimma, Ethiopia

**Keywords:** Semilinear singularly perturbed, Fourth-order, Non-uniform mesh, Accurate solution, 34B08, 34D15, 34D20, 65L11, 65L50

## Abstract

**Objective:**

The main purpose of this work is to present a fourth-order fitted mesh scheme for solving the semilinear singularly perturbed reaction–diffusion problem to produce more accurate solutions.

**Results:**

Quasilinearization technique is used to linearize the semilinear term. The scheme is formulated with discretizing the solution domain piecewise uniformly and then replacing the differential equation by finite difference approximations. This gives the system of difference algebraic equations and is solved by the Thomas algorithm. Convergence analysis are investigated using solution bound and the truncation error bound. Numerical illustrations are investigated to support the theoretical results and the method’s applicability. The method produces a more accurate solution than some existing methods in the literature.

## Introduction

Any differential equation in which the coefficient of highest order derivative is a small positive parameter together with the prescribed conditions is called singularly perturbed problem, [1]. This problem arise very frequently in diversified fields of applied mathematics and engineering; for instance fluid mechanics, elasticity, hydrodynamics, quantum mechanics, chemical-reactor theory, aerodynamics, plasma dynamics, modeling of semiconductor devices, diffraction theory and reaction–diffusion processes and many other allied areas [2–8]. Hence, due to the importance of these problems in real life situations, the need to develop numerical methods for approximation its solution is advantageous.

Singularly perturbed problems broadly categorized into reaction–diffusion and convection–diffusion types, [9–15, 22]. Thes can be further classified according to the type of layers (boundary and/or interior layers), location (left or right or twin), type of defined conditions like initial or boundary (Dirichlet, robin or mixed conditions). More particularly, the singularly perturbed reaction–diffusion boundary valued problems are categorized into linear and nonlinear problems exhibiting twin (both left and right) boundary layers. These types of problems occur frequently in fluid mechanics and other several fields of applied mathematics, [16–22].

As both books in [10, 15], explain, the region at which the solution of singularly perturbed problems change very quickly in certain small regions called layer region. It is well known that when the perturbation parameter is small enough, classical numerical methods fails to produce the required accurate solution for these problems. Thus, it should be important to develop appropriate numerical methods for such problems. There are several numerical methods suggested by various authors for solving the families of the linear singularly perturbation reaction–diffusion problems. Some and most of them are higher order (fourth, sixth, eighth, and tenth order) compact finite difference methods to solve different types singularly perturbed reaction–diffusion problems [2–5]. Most authors in these literatures developed the methods by restricting the criteria for the constant coefficients of the reaction term within the problem. Further, scholars in [2, 13], have presented fourth and sixth-order stable central difference method, respectively, for solving singularly perturbed two-point boundary value problem. This type of problem shares the basic behavior of singularly perturbed reaction–diffusion boundary value problem. Authors in [23] presented a numerical approach to solving singularly perturbed semilinear convection–diffusion problems. The nonlinear part of the problem is linearized via the quasilinearization technique.

Recently, a numerical scheme for solving the semilinear singularly perturbed reaction–diffusion problems and a numerical technique for solving a class of nonlinear singularly perturbed boundary value problems have been presented respectively, in [2, 14]. They have introduced a basic and computational approach scheme based on Numerov’s type on uniform mesh. They indicated that the method is uniformly convergence, according to the discrete maximum norm, independently of the perturbation parameter. Also, authors in [3], deliberate a numerical scheme based on Sinc collocation method to solve a class of nonlinear singularly perturbed boundary value problems which exhibit twin boundary layers. The Sinc method can control the oscillations in computed solutions at boundary layer regions naturally because the distribution of Sinc points is denser at near the boundaries. The numerical results support the theoretical results and illustrate the validity and accuracy of the method compared with the results in the existing methods. So far different finite difference methods have been adopted for solving the semilinear singularly perturbed reaction–diffusion problems. However, the obtained accurate solution and the existing rate of convergence are attracts remarkable attention to be improve. Thus, the main drawbacks to most of the presented methods are using uniform mesh of the solution domain, restricting the coefficient reaction term to constant function only, the method gives accurate solution when the mesh size of the solution domain and the values of the perturbation parameter are comparable. Moreover, most presented numerical schemes for solving the considered problem are limited to the second order convergent.

Therefore, in this paper, the main objective is to present a fourth-order fitted mesh scheme that works for variable coefficient of the reaction term for solving the semilinear singularly perturbed boundary value problems. Accordingly, in this effort, we have been formulated, analyze and implement the fourth-order fitted mesh scheme that produces a more accurate solution for solving semilinear singularly perturbed reaction–diffusion problems.

## Main text

### Description of the method

This paper deals with singularly perturbed semilinear reaction–diffusion problem:

1 $$ - \varepsilon y^{\prime\prime}(x) + q(x)y(x) + g(x,y(x)) = 0,\quad \forall x \in (0,1), $$

subject to the conditions

2 $$ y(0) = A,\quad y(1) = B, $$

where $\varepsilon ,\,\,\,0 < \varepsilon < < 1$ is perturbation parameter, $A$ and $B$ are given constant numbers. Assume that the functions $q(x)$ and the nonlinear term $g(x,y(x))$ is given sufficiently smooth with $g(x,y(x)) \in C[0,1],$ [0,1], and

3 $$ q(x) + \frac{\partial g(x,y(x))}{{\partial y}} \ge \beta > 0,\quad \forall x \in [0,1], $$

for some constant $\beta > 0,$ to ensure the existence and unique solution with dual boundary layers near the two end points of the solution domain [14]. The reduced problem of Eq. (1) is obtained by setting $\varepsilon = 0,$ gives:

4 $$ q(x)y(x) + g(x,y(x)) = 0\,. $$

With these conditions Eqs. (1) and (2) and the reduced problem in Eq. (4) have a unique solution. The unique solution to Eqs. (1) and (2) exhibits two boundary layers at the end of the interval $\overline{\Omega } = [0,\,1]$, as the perturbation parameter $\varepsilon$ approaches to zero [2, 20, 22].

Quasilinearization technique used to transform the semilinear singularly perturbed reaction–diffusion problem into a sequence of linear equations, [14]. We choose a reliable initial approximation for the function $y^{(0)} (x)$ in $g(x,y(x))$ as:

5 $$ y^{(0)} (x) = mx + b,\, $$

where $m$ and $b$ are arbitrary constants determined using Eq. (2). Hence, Eq. (5) becomes:

6 $$ y^{(0)} (x) = (B - A)x + A. $$

By Taylor series expansion, we expand $g(x,y(x))$ around the chosen initial approximation:

7 $$ g(x,y^{(k + 1)} (x)) \simeq g(x,y^{(k)} (x)) + (y^{(k + 1)} - y^{k} )\frac{{\partial g^{(k)} }}{\partial y}\,|_{{(x,y^{(k)} (x))}} + \,\,...\,, $$

where $k = 0,1,2,...$ is the number of iterations index. Substituting Eq. (7) into Eq. (1) and (2), we have:

8 $$ \begin{aligned} - \varepsilon y^{\prime \prime (k + 1)} (x) & + q(x)y^{(k + 1)} (x) + \frac{{\partial g^{(k)} }}{\partial y}|_{{(x,y^{(k)} (x)}} y^{(k + 1)} (x) \hfill \\ & = - g(x,y^{(k)} (x)) + y^{(k)} (x)\frac{{\partial g^{(k)} }}{\partial y}|_{{_{{(x,y^{(k)} (x)}} }} + \cdots \,\,, \hfill \\ \end{aligned} $$

subject to the boundary conditions

9 $$ y^{(k + 1)} (0) = A,\,\,\,\,\,\,\,y^{(k + 1)} (1) = B\, $$

From now, Eq. (8) is linear in $y^{(k + 1)} (x).$ Thus, we solve the sequence of Eq. (8) in place of the semilinear problem in Eq. (1) by fourth-order fitted mesh scheme, which will be introduced in the next section. For the solution of the semilinear boundary value problem, we require that

10 $$ \mathop {\max \,}\limits_{k \to \infty } y^{(k)} (x) = y^{(*)} (x),\,\, $$

where $y^{(*)} (x)$ is the solution of the semilinear problem. Numerically, we require that:

11 $$ |y^{(k + 1)} (x) - y^{(k)} (x)|\,\, < \,\lambda ,\, $$

where $\lambda$ is a small tolerance chosen by us. Then $y^{(k + 1)} (x)$ is the approximate solution of the semilinear problem.

### Properties of continuous problem

For the sake of simplicity, at first iteration or $(k = 0)$, let us denote Eqs. (8) and (9) in the form of:

12 $$ Lu(x) = - \varepsilon u^{\prime\prime}(x) + p(x)u(x) = f(x),\,\,\,x \in \Omega : = (0,1)\,, $$

where the coefficient of reaction term and the source terms are respectively:

$p(x) = q(x) + \frac{\partial g(x,y(x))}{{\partial y}}|_{{(x,y^{(0)} (x))}} \, \ge \,\,\beta > 0,$ and $f(x) = - g(x,y^{(0)} (x)) + y^{(0)} (x)\frac{\partial g(x,y(x))}{{\partial y}}|_{{(x,y^{(0)} (x))}} ,$and $y^{(k + 1)} (x) = u(x)$ with the operator $L$, such that:

13 $$ u(0) = A,\,\,\,\,\,\,u(1) = B\,. $$

We present some important properties for the solution of Eqs. (12) and (13) which will be useful in the subsequent section for the analysis of relevant numerical solutions.

#### **Lemma 1**

 (Continuous Maximum Principle), [14], *Assume that* $v(x)$ *is sufficiently smooth function which satisfies* $v(0) \ge 0$* and* $v(1) \ge 0$. *Then* $Lv(x) \ge 0,\,\,\,\,0 < x < 1,$ *implies that* $v(x) \ge 0$ $\forall x \in [0,1].$

#### ***Proof***

Let $v$ be a value such that $v(x^{*} ) = \mathop {\min }\limits_{x \in \Omega } v(x),$ and assume that $v(x^{*} ) < 0.$

Clearly, $x^{*} \notin \{ 0,1\} ,$ and therefore, $v^{\prime}(x^{*} ) = 0$ and $v^{\prime\prime}(x) \ge 0.$ Moreover, there is

$$ Lv(x^{*} ) = - \varepsilon v^{\prime\prime}(x^{*} ) + p(x)v(x) < 0 $$

which is a contradiction. It follows that $v(x^{*} ) \ge 0$ and thus, $v(x) \ge 0,\,\,\,\forall x \in \Omega$.$\square$

#### **Lemma 2**

 (Uniform Stability Estimate), [14], *Let* $u(x)$ *be the solution of Eqs. (4.12) and (4.13). Then, we have:*

$$ ||u(x)||\,\, \le \,\,\beta^{ - 1} ||f||\, + \max (|A|,|B|),\,\,\,\forall x \in [0,1]. $$

#### ***Proof***

 We construct two barrier functions $\psi^{ \pm }$ defined by.

$$ \psi^{ \pm } = \beta^{ - 1} ||f|| + \max (|A|,|B|) \pm u(x) $$

Then it can be said that

$$ \begin{gathered} \psi^{ \pm } (0) = \beta^{ - 1} ||f|| + \max (|A|,\,|B|) \pm u(0) = \beta^{ - 1} ||f|| + \max (|A|,\,|B|) \pm A \ge 0; \hfill \\ \psi^{ \pm } (1) = \beta^{ - 1} ||f|| + \max (|A|,\,|B|) \pm u(1)\, = \beta^{ - 1} ||f|| + \max (|A|,\,|B|) \pm B\, \ge 0; \hfill \\ \end{gathered} $$

It follows that

$$ \begin{aligned} L\psi^{ \pm } (x) & = - \varepsilon (\psi^{ \pm } (x))^{\prime\prime} + p(x)\psi^{ \pm } (x) \hfill \\ & = - \varepsilon [\beta^{ - 1} ||f|| + \max (|A|,|B|) \pm u(x)]^{\prime\prime} + p(x)[\beta^{ - 1} ||f|| + \max (|A|,|B|) \pm u(x)] \hfill \\ & = \pm ( - \varepsilon u^{\prime\prime}(x)) \pm p(x)u(x) + p(x)[\beta^{ - 1} ||f|| + \max (|A|,|B|)] \hfill \\ \,\,\,\,\,\,\,\,\,\,\,\,\,\,\,\,\,\, \hfill \\ \end{aligned} $$

Because, $$[\beta^{ - 1} ||f|| + \max (|A|,|B|)]^{\prime\prime} = 0$$

$$ \begin{aligned} L\psi^{ \pm } (x) & = \pm [ - \varepsilon u^{\prime\prime}(x)) + p(x)u(x)] + p(x)[\beta^{ - 1} ||f|| + \max (|A|,|B|)] \hfill \\ &  = \pm Lu(x) + p(x)[\beta^{ - 1} ||f|| + \max (|A|,|B|)] \hfill \\ &  = p(x)[\beta^{ - 1} ||f|| + \max (|A|,|B|)] \pm Lu(x) \hfill \\ \end{aligned} $$

Since, $$p(x) \ge \beta > 0,\,\,\,\,\,||f||\, \ge \,f(x),\,\,\,\,{\text{and}}\,\,\,\,Lu(x) = f(x)$$

$$ L\psi^{ \pm } (x) = p(x)[\beta^{ - 1} ||f|| + \max (|A|,|B|)] \pm f(x)\,\,\, \ge \,\,0. $$

Therefore, $L\psi^{ \pm } (x) \ge 0$. Thus, using Lemma 1, we get $\psi^{ \pm } (x) \ge 0,\,\,\,\,\forall x \in [0,1]$ this gives the required estimate. Further, we derive the bounds for the solution $u(x)$ and its derivative by the following estimate. $\square$

#### **Lemma 3**

 (Boundedness), [14], *Let* $u(x)$ *be the solution of Eqs. (4.12) and (4.13), with* $p(x)$ *and* $f(x)$ *are given sufficiently smooth functions. Then the following estimates hold.*

14 $$ ||u(x)||_{\infty } \le C,\,\,\,\,\,0 \le x \le 1 $$

15 $$ |u^{\prime}(x)|\,\, \le C\left\{ {1 + \frac{1}{\sqrt \varepsilon }\left( {e^{{ - \sqrt {\frac{\alpha }{\varepsilon }} x}} + e^{{ - \sqrt {\frac{\alpha }{\varepsilon }} (1 - x)}} } \right)} \right\}\, $$

#### ***Proof***

 Applying Lemma 2, to Eqs. (12) and (13), we have Eq. (14).

16 $$ Lv(x) = \phi (x). $$

17 $$ v(0) = O\left( {\frac{1}{\sqrt \varepsilon }} \right) = v(1), $$

where $$v(x) = u^{\prime}(x).$$

18 $$ \phi (x) = f^{\prime}(x) - p^{\prime}(x)u(x). $$

The solution of the problem in Eq. (16) and (17) has the following forms:

19 $$ v(x) = v_{0} (x) + v_{1} (x) $$

where the functions $v_{0} (x)$ and $v_{1} (x)$ are the solutions of the following problems respectively

20 $$ \left\{ \begin{gathered} Lv_{0} (x) = \phi (x),\,\,\,\,\,\,0 < x < 1, \hfill \\ v_{0} (0) = v_{1} (1) = 0,\,\,\,\,\,\,\,\,\,\,\,\,\,\,\,\,\,\,\,\,\,\,\,\,\,\,\,\,\,\,\,\,\,\,\,\,\,\,\,\,\,\,\,\,\,\,\,\, \hfill \\ \end{gathered} \right. $$

and

21 $$ \left\{ \begin{gathered} Lv_{1} (x) = 0,\,\,\,0 < x < 1, \hfill \\ v_{0} (0) = v_{1} (1) = 0,\,\,\,\,\,\,\,\,\,\,\,\,\,\,\, \hfill \\ \end{gathered} \right. $$

Using Lemma 2, for the solution of the problem Eq. (20), we have:

$$ |v_{0} (x)|\,\, \le \,\beta^{ - 1} \mathop {\max }\limits_{0 \le s \le 1} |\phi (s)|\,\, $$

Thus, we obtain:

22 $$ |v_{0} (x)|\,\, \le \,\,C,\,\,\,\,\,0 \le x \le 1 $$

Applying the maximum principle to the problem Eq. (21), gives

23 $$ |v_{1} (x)|\,\, \le w(x)\,\,\, $$

where $w(x)$ is the solution of the following problem:

24 $$ \left\{ \begin{gathered} - \varepsilon w^{\prime\prime}(x) + \beta w(x) = 0,\,\,\,0 < x < 1, \hfill \\ w(0) = |v_{1} (0)|,\,\,\,w(1) = |v_{1} (1)| \hfill \\ \end{gathered} \right. $$

The solution of Eq. (4.24) has the form:

25 $$ w(x) \le C\left\{ {\frac{1}{\sqrt \varepsilon }\left( {e^{{ - \,\,\sqrt {\frac{\beta }{\varepsilon }} x}} + e^{{ - \sqrt {\frac{\beta }{\varepsilon }} (1 - x)}} } \right)} \right\}\, $$

Then, combining Eqs. (22), (23) and (25) we get the inequality,

$|u^{\prime}(x)|\,\, \le \,\,|v_{0} (x)| + |v_{1} (x)|\,\, \le C + w(x)\, = C + \frac{C}{\sqrt \varepsilon }\left( {e^{{ - \sqrt {\frac{\beta }{\varepsilon }} x}} + e^{{ - \sqrt {\frac{\beta }{\varepsilon }} (1 - x)}} } \right).$ Thus, the proof is completed. $\square$

### Mesh generation

We construct a non-uniform mesh that contains more number of nodal points in the layer regions than non-layer region. The domain $[0,1]^{N} ,\,\,N \ge 4$ is divided into three subintervals, $[0,\tau ],\,\,[\tau ,1 - \tau ],\,\,[1 - \tau ,1]$ where the chosen transition parameter,

26 $$ \tau = \min \left\{ {\frac{1}{4},\sqrt \varepsilon \ln (1/\sqrt \varepsilon )} \right\}, $$

denotes the width of the boundary layers. The domain $[0,1]^{N}$ is obtained by putting a non-uniform mesh with $\frac{N}{4}$ mesh elements in both the layer intervals and a uniform mesh with $\frac{N}{2}$ mesh elements in the outer layer region.

A general non-uniform mesh $[0,1]^{N} = \left\{ {0 = x_{0} ,x_{1} ,x_{2} ,...,x_{N} = 1} \right\}$ with step size will be defined as

27 $$ h_{i} = x_{i} - x_{i - 1} = \left\{ \begin{aligned} & \frac{4\tau }{N},\,\,i = 1,2,...,\frac{N}{4} \hfill \\ & \frac{2(1 - 2\tau )}{N},\,\,i = \frac{N}{4} + 1,...,\frac{3N}{4}, \hfill \\ & \frac{4\tau }{N},\,\,i = \frac{3N}{4} + 1,...,N. \hfill \\ \end{aligned} \right.\,\,\, $$

### Formulation of the scheme

In order to formulate the scheme, consider the linear singularly perturbed differential equation in Eq. (12) subject to the conditions in Eq. (13) that gives the boundary value problem:

28 $$ \left\{ \begin{aligned}& - \varepsilon u^{\prime\prime}(x) + p(x)u(x) = f(x),\quad 0 < x < 1, \hfill \\ & u(0) = A, \hfill \\ & u(1) = \,B. \hfill \\ \end{aligned} \right. $$

Let us define the three the nodal points based finite difference approximation from the general multistep finite difference for the differential equation part as in the form:

29 $$ \sum\limits_{j = 0}^{2} {a_{j} U_{i - j + 1} = \sum\limits_{j = 0}^{2} {b_{j} U^{\prime\prime}_{i - j + 1} ,} } $$

where the coefficient parameters $a_{j}$ and $b_{j}$ are determined in terms the mesh parameter $h_{i}$. These parameters one can obtain in a similar way as on equidistant mesh. Hence, let the local truncation error estimated from Eq. (29) written as:

30 $$ T_{i} = a_{0} U_{i - 1} + a_{1} U_{i} + a_{2} U_{i + 1} - [b_{0} U^{\prime\prime}_{i - 1} + b_{1} U^{\prime\prime}_{i} + b_{2} U^{\prime\prime}_{i + 1} ]. $$

Assume that the function $u(x)$ has continuous derivatives of sufficiently fourth-order. Expanding the terms $U(x_{i \pm 1} )$ and $U^{\prime\prime}(x_{i \pm 1} )$ by Taylor’s series expansion about the point $x_{i}$ as:

31 $$ \left\{ \begin{aligned} & U_{i + 1} = U_{i} + h_{i + 1} U^{\prime}_{i} + \frac{{h_{i + 1}^{2} }}{2!}U^{\prime\prime}_{i} + \frac{{h_{i + 1}^{3} }}{3!}U^{\prime\prime\prime}_{i} + \frac{{h_{i + 1}^{4} }}{4!}U_{i}^{(4)} + \frac{{h_{i + 1}^{5} }}{5!}U_{i}^{(5)} + \frac{{h_{i + 1}^{6} }}{6!}U_{i}^{(6)} + O(h_{i + 1}^{7} ) \hfill \\ & U_{i - 1} = U_{i} - h_{i} U^{\prime}_{i} + \frac{{h_{i}^{2} }}{2!}U^{\prime\prime}_{i} - \frac{{h_{i}^{3} }}{3!}U^{\prime\prime\prime}_{i} + \frac{{h_{i}^{4} }}{4!}U_{i}^{(4)} - \frac{{h_{i}^{5} }}{5!}U_{i}^{(5)} + \frac{{h_{i}^{6} }}{6!}U_{i}^{(6)} + O(h_{i}^{7} ) \hfill \\ & U^{\prime\prime}_{i + 1} = U^{\prime\prime}_{i} + h_{i + 1} U^{\prime\prime\prime}_{i} + \frac{{h_{i + 1}^{2} }}{2!}U_{i}^{(4)} + \frac{{h_{i + 1}^{3} }}{3!}U_{i}^{(5)} + \frac{{h_{i + 1}^{4} }}{4!}U_{i}^{(6)} + O(h_{i + 1}^{7} ) \hfill \\ & U^{\prime\prime}_{i - 1} = U^{\prime\prime}_{i} - h_{i} U^{\prime\prime\prime}_{i} + \frac{{h_{i}^{2} }}{2!}U_{i}^{(4)} - \frac{{h_{i}^{3} }}{3!}U_{i}^{(5)} + \frac{{h_{i}^{4} }}{4!}U_{i}^{(6)} + O(h_{i}^{7} ) \hfill \\ \end{aligned} \right.\, $$

Then, substituting this Eq. (31) into Eq. (30) and grouping like terms gives:

32 $$ \begin{aligned} T_{i} & = (a_{0} + a_{1} + a_{2} )U_{i} + (a_{0} h_{i + 1} - a_{2} h_{i} )U^{\prime}_{i} \hfill \\ & \quad + (\frac{{a_{0} h_{i + 1}^{2} }}{2!} + \frac{{a_{2} h_{i}^{2} }}{2!} - (b_{0} + b_{1} + b_{2} ))U_{i}^{\prime \prime } \hfill \\ & \quad + (\frac{{a_{0} h_{i + 1}^{3} }}{3!} - \frac{{a_{2} h_{i}^{3} }}{3!} - (b_{0} h_{i + 1} - b_{2} h_{i} ))U^{\prime\prime\prime}_{i} \hfill \\ & \quad + (\frac{{a_{0} h_{i + 1}^{4} }}{4!} + \frac{{a_{2} h_{i}^{4} }}{4!} - (\frac{{b_{0} h_{i + 1}^{2} }}{2!} + \frac{{b_{2} h_{i}^{2} }}{2!}))U_{i}^{(4)} \hfill \\ & \quad + (\frac{{a_{0} h_{i + 1}^{5} }}{5!} - \frac{{a_{2} h_{i}^{5} }}{5!} - (\frac{{b_{0} h_{i + 1}^{3} }}{3!} - \frac{{b_{2} h_{i}^{3} }}{3!}))U_{i}^{(5)} \hfill \\ & \quad + (\frac{{a_{0} h_{i + 1}^{6} }}{6!} + \frac{{a_{2} h_{i}^{6} }}{6!} - (\frac{{b_{0} h_{i + 1}^{4} }}{4!} + \frac{{b_{2} h_{i}^{4} }}{4!}))U_{i}^{(6)} + ...\, \hfill \\ \end{aligned} $$

The method given in Eq. (29), is of order four if all the coefficients given in Eq. (32) are equal to zero except it is differ from zero after the coefficient of $U_{i}^{(6)} ,$ which gives the system of equation:

33 $$ \begin{aligned} & \left\{ \begin{aligned} & a_{0} + a_{1} + a_{2} = 0 \hfill \\ & a_{0} h_{i + 1} - a_{2} h_{i} = 0 \hfill \\ & a_{0} h_{i + 1}^{2} + a_{2} h_{i}^{2} = 2(b_{0} + b_{1} + b_{2} ) \hfill \\ & a_{0} h_{i + 1}^{3} - a_{2} h_{i}^{3} = 6(b_{0} h_{i + 1} - b_{2} h_{i} ) \hfill \\ & a_{0} h_{i + 1}^{4} + a_{2} h_{i}^{4} = 12(b_{0} h_{i + 1}^{2} + b_{2} h_{i}^{2} ) \hfill \\ & a_{0} h_{i + 1}^{5} - a_{2} h_{i}^{5} = 20(b_{0} h_{i + 1}^{3} - b_{2} h_{i}^{3} ) \hfill \\ &  \end{aligned} \right.\, \hfill \\ & \quad a_{0} h_{i + 1}^{6} + a_{2} h_{i}^{6} \ne 30(b_{0} h_{i + 1}^{4} + b_{2} h_{i}^{4} ) \hfill \\ \end{aligned} $$

Adapting $b_{0} + b_{1} + b_{2} = 1,$ from the relation in uniform mesh, the solution of the system in Eq. (33) is determined by using the usually elimination method gives:

34 $$ \left\{ \begin{aligned} & a_{0} = \frac{2}{{h_{i + 1} (h_{i} + h_{i + 1} )}} \hfill \\ & a_{1} = \frac{ - 2}{{h_{i} h_{i + 1} }} \hfill \\ & a_{2} = \frac{2}{{h_{i} (h_{i} + h_{i + 1} )}} \hfill \\ \end{aligned} \right.\,\,\,\,\,\,\,\,\,\,and\,\,\,\,\,\,\,\,\,\,\, \left\{ \begin{aligned} & b_{0} = \frac{{h_{i + 1}^{2} + h_{i} h_{i + 1} - h_{i}^{2} }}{{6h_{i + 1} (h_{i} + h_{i + 1} )}} \hfill \\ & b_{1} = \frac{{4h_{i} h_{i + 1} (h_{i} + h_{i + 1} ) + h_{i}^{3} + h_{i + 1}^{3} }}{{6h_{i} h_{i + 1} (h_{i} + h_{i + 1} )}} \hfill \\ & b_{2} = \frac{{h_{i} h_{i + 1} + h_{i}^{2} - h_{i + 1}^{2} }}{{6h_{i} (h_{i} + h_{i + 1} )}} \hfill \\ \end{aligned} \right.\,\, $$

Using this we approximate the problem in Eq. (28) by Eq. (29), which can write in the form of:

35 $$ a_{0} U_{i - 1} + a_{1} U_{i} + a_{2} U_{i + 1} = b_{0} U^{\prime\prime}_{i - 1} + b_{1} U^{\prime\prime}_{i} + b_{2} U^{\prime\prime}_{i + 1} ,\,\, $$

Then, considering from the differential equation in Eq. (28), at the nodal point $x_{i} ,$ we have:

36 $$ U^{\prime\prime}_{i} = \frac{{p_{i} U_{i} - f_{i} }}{\varepsilon },\,\,\,\,\,U^{\prime\prime}_{i - 1} = \frac{{p_{i - 1} U_{i - 1} - f_{i - 1} }}{\varepsilon },\,\,\,\,\,\,{\text{and}}\,\,\,U^{\prime\prime}_{i + 1} = \frac{{p_{i + 1} U_{i + 1} - f_{i + 1} }}{\varepsilon }. $$

Substituting Eq. (36) into Eq. (35) and also using the values in Eq. (34), we obtain the three-term recurrence finite difference scheme

37 $$ E_{i} U_{i - 1} + F_{i} U_{i} + G_{i} U_{i + 1} = H_{i} ,\,\,\,\,\,\,\,\,i = 1,2,\,\,...,\,\,N - 1,\,\, $$

where

$$ \begin{aligned} E_{i} & = \frac{ - 2\varepsilon }{{h_{i + 1} (h_{i + 1} + h_{i} )}} + \frac{{p_{i - 1} (h_{i + 1}^{2} + h_{i} h_{i + 1} - h_{i}^{2} )}}{{6h_{i + 1} (h_{i + 1} + h_{i} )}},\quad F_{i} = \frac{2\varepsilon }{{h_{i} h_{i + 1} }} + \frac{{P_{i} (4h_{i} h_{i + 1} (h_{i + 1} + h_{i} ) + h_{i}^{3} + h_{i + 1}^{3} )}}{{6h_{i} h_{i + 1} (h_{i + 1} + h_{i} )}} \hfill \\ G_{i} & = \frac{ - 2\varepsilon }{{h_{i} (h_{i + 1} + h_{i} )}} + \frac{{p_{i + 1} (h_{i}^{2} + h_{i} h_{i + 1} - h_{i + 1}^{2} )}}{{6h_{i} (h_{i + 1} + h_{i} )}}, \hfill \\ H_{i} & = \left( {\frac{{h_{i + 1}^{2} + h_{i} h_{i + 1} - h_{i}^{2} }}{{6h_{i + 1} (h_{i + 1} + h_{i} )}}} \right)f_{i - 1} + \left( {\frac{{4h_{i} h_{i + 1} (h_{i + 1} + h_{i} ) + h_{i}^{3} + h_{i + 1}^{3} }}{{6h_{i} h_{i + 1} (h_{i + 1} + h_{i} )}}} \right)f_{i} + \left( {\frac{{h_{i}^{2} + h_{i} h_{i + 1} - h_{i + 1}^{2} }}{{6h_{i} (h_{i + 1} + h_{i} )}}} \right)f_{i + 1} . \hfill \\ \end{aligned} $$

Considering Eq. (4.2) and by solving this system of linear algebraic equations we obtain the approximate solution $U_{i} ,\,i = 0,1,2,...,N$ of $u(x_{i} )$ at the nodal points $x_{0} ,x_{1} ,x_{2} ,...,x_{N} .$

### Convergence analysis

Let $u_{i}$ be the solution of Eq. (12) and $U_{i}$ be the solution to Eq. (37) at the nodal point $x_{i}$, then $z_{i} = u_{i} - U_{i}$,$0 \le i \le N$, with the estimate approximate error $z_{i}$, which satisfies the discrete problem

38 $$ \left\{ \begin{aligned} & Lz_{i} = R_{i} , \hfill \\ & z_{0} = 0 = z_{N,} \hfill \\ \end{aligned} \right. $$

where $R_{i}$ is the truncation error in Eq. (32).

#### **Lemma 4**

 (Discrete Maximum Principle): *Suppose that a mesh function* $v_{i}$ *satisfies* $v_{0} \ge 0$ *and* $v_{N} \ge 0$. *Then* $L^{N} v_{i} \ge 0,$ $\forall \,\,\,1 \le i \le N - 1$ *implies that* $v_{i} \ge 0$, $\forall \,\,\,0 \le i \le N$.

#### ***Proof***

 Let $V_{i}$ be a value such that $V_{i}^{*} = \mathop {\min \,v_{i} }\limits_{1 \le i \le N - 1}$ and assume that $V_{i}^{*} < 0.$ Clearly, $i \in \left\{ {0,N} \right\}$ and therefore $V_{i}^{*\prime } = 0$ and $V_{i}^{*\prime \prime } \ge 0.$ Moreover, there is.

$$ \begin{gathered} L^{N} V_{i} = a_{0} V_{i + 1} + a_{1} V_{i} + a_{2} V_{i - 1} - [b_{0} V^{\prime\prime}_{i + 1} + b_{1} V^{\prime\prime}_{i} + b_{2} V^{\prime\prime}_{i - 1} ], \hfill \\ L^{N} V_{i} = a_{0} V_{i + 1} + a_{1} V_{i} + a_{2} V_{i - 1} - [b_{0} V^{\prime\prime}_{i + 1} + b_{1} V^{\prime\prime}_{i} + b_{2} V^{\prime\prime}_{i - 1} ] < 0. \hfill \\ \end{gathered} $$

which is a contradiction. It follows that $V_{i}^{*} \ge 0$ and thus $v_{i} \ge 0,\,\,\forall \,\,\,0 \le i \le N$.$\square$

#### **Lemma 5**

 (Uniform Stability Estimate): *If *$U_{i}$ *is any mesh function such that* $U_{i} = 0 = U_{N}$, *then*

39 $$ |U_{i} |\, \le \beta^{ - 1} \mathop {\max }\limits_{1 \le i \le N - 1} |L^{N} U_{i} |,\,\,\,0 \le i \le N $$

#### ***Proof***

 Denote $Z_{i} = \beta^{ - 1} \mathop {\max }\limits_{1 \le i \le N - 1} |L^{N} U_{i} |,\,\,\,1 \le i \le N - 1.$ Introduce two mesh functions

$$\xi_{i}^{ \pm } = Zp_{i} \pm U_{i}$$, Clearly, $$\xi_{0}^{ \pm } = 0 = \xi_{N}^{ \pm }$$ and $$\forall \,\,1 \le i \le N - 1$$:

$$ L^{N} \xi_{i}^{ \pm } = Zp_{i} + L^{N} U_{i} \le 0. $$

Since $p_{i} \ge \beta \ge 0$ Lemma 4 implies that $\xi_{i}^{ \pm } \ge 0,\,\,\forall \,\,0 \le i \le N,$ and this completes the proof. $\square$

#### **Lemma 6**

 (Error Boundness), *The truncation error at the grid point* $x_{i}$ *is given by:*

40 $$ L^{*} (u - U)_{i} = Lu_{i} - L^{N} U_{i} $$

*From the formulated method, the non-zero estimated local truncation error provided in Eq. (*32*) with the conditions in Eqs. (*33*) and (*34*) written as:*

41 $$ T_{i} = \left( {\frac{{a_{0} h_{i + 1}^{6} }}{6!} + \frac{{a_{2} h_{i}^{6} }}{6!} - \left( {\frac{{b_{0} h_{i + 1}^{4} }}{4!} - \frac{{b_{2} h_{i}^{4} }}{4!}} \right)} \right)U_{i}^{(6)} $$

*where* $a_{0} ,\,a_{2} ,\,b_{0} ,\,b_{2}$ *are defined in Eq. (*34*)*.

*Thus, from the relation* $h_{i}^{4} > h_{i}^{6}$*, we have*

42 $$ |T_{i} |\, = \,|L^{*} (u - U)_{i} |\,\, \le \,\,C(h_{i}^{4} + h_{i + 1}^{4} ) $$

*for* $C = \frac{1}{24}\left\{ {||b_{0} ||_{\infty } + ||b_{2} ||_{\infty } } \right\}||U_{i}^{(6)} ||_{\infty }$ *is arbitrary constant.*

*Let us consider* $h = \mathop {\max }\limits_{\forall \,\,i \to \infty } \left\{ {h_{i} ,\,\,h_{i + 1} } \right\}$ *and applying the uniform stability estimate (Lemma 5), yields:*

43 $$ \mathop {\max }\limits_{0 \le i \le N} |u - U|\,\, \le Ch^{4} $$

*Hence, the formulated method is fourth order convergent.*

### Numerical examples and results

To illustrate the applicability of the proposed method, we applied it model example. For the example whose exact solution is unknown, we use the double mesh principle to estimate the error and compute the experimental rate of convergence. The double mesh principle is given by:

44 $$ D_{\varepsilon }^{N} = \mathop {\max }\limits_{{x_{i} \in [0,1]^{N} }} |U_{i}^{N} - U_{i}^{2N} |,\,\, $$

where $U_{i}^{N}$ and $U_{i}^{2N}$ respectively, denotes the numerical solution obtained using *N* and *2N* mesh intervals. Further, we calculate the order of convergence by the formula:

45 $$ R = \frac{{\log (D_{\varepsilon }^{N} ) - \log (D_{\varepsilon }^{2N} )}}{\log (2)}.\,\,\,\, $$

#### **Example 1**

 Consider the semilinear singularly perturbed reaction–diffusion problem, [14]:

$$ \left\{ \begin{aligned} & - \varepsilon y^{\prime\prime}(x) - e^{{ - (x^{2} + y)}} = 0, \quad 0 < x < 1, \hfill \\ & y(0) = 0, \hfill \\ & y(1) = 1. \hfill \\ \end{aligned} \right. $$

The exact solution for this problem is unknown.

#### **Example 2**

 Consider the linear singularly perturbed problem.

$$ \left\{ \begin{gathered} - \varepsilon y^{\prime\prime}(x) + (1 + x(1 - x))y(x) = f(x),\,\,\,\,\,\,\,\,0 < x < 1, \hfill \\ \,\,\,\,y(0) = 0, \hfill \\ \,\,\,y(1) = 0, \hfill \\ \end{gathered} \right. $$.

The source function is given by:

$$ \begin{aligned} f(x) &= 1 + x(1 - x) + (2\sqrt \varepsilon - x^{2} + x^{3} )\exp (\frac{ - (1 - x)}{{\sqrt \varepsilon }}) \hfill \\ & \quad + (2\sqrt \varepsilon - x(1 - x)^{2} )\exp (\frac{ - x}{{\sqrt \varepsilon }}) \hfill \\ \end{aligned} $$.

The exact solution is $y(x) = 1 + (x - 1)\exp (\frac{ - x}{{\sqrt \varepsilon }}) - x\exp (\frac{ - (1 - x)}{{\sqrt \varepsilon }}).$

## Conclusion

**Fig. 1:**
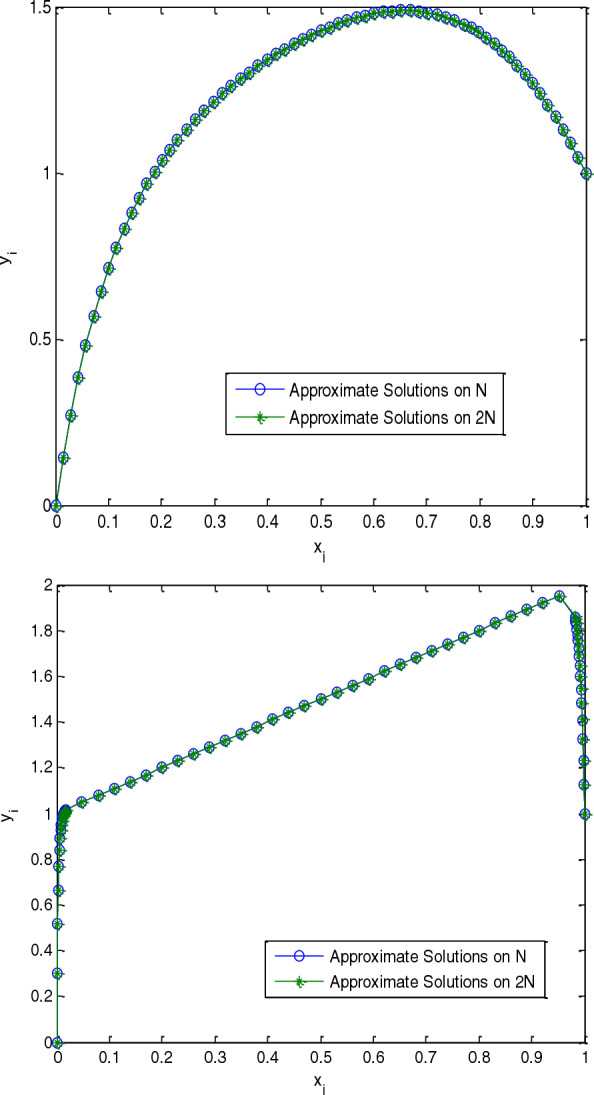
Numerical solution for Example 1 with $N = 64$, $\varepsilon = 10^{ - 2}$ and $\varepsilon = 10^{ - 5}$ respectively

In this paper, fourth-order fitted mesh scheme is presented for solving semilinear singularly perturbed reaction–diffusion problem. From this problem, the nonlinear part is linearized by the quasilinearization technique. The convergence analysis of the described method established theoretically as well as confirmed in numerical illustration that is fourth order convergent. To validate the applicability of method, model examples are considered and numerical results investigated in tabular and graphic forms. Specifically, results are expressed in terms of maximum absolute errors, and rate of convergence. The result in Table 1 indicates that the comparison of maximum absolute errors for the proposed method and the methods in [14]. Further, we observe that as the number mesh increases the maximum absolute error decreases in each row implying that the proposed method is convergent. Tables 2 and 3 indicates that the rate of convergence for the described method is fourth-order convergent. This confirms the theoretical investigations given by Eq. (43). Also, Fig. 1 used to visualize the effect of perturbation parameter and the boundary layer behaviors. Furthermore, the main originality of the suggested method describe and examined in terms the obtained more accurate solutions with higher order of convergence as our original contributions. Generally, the method is fourth-order convergent and gives more accurate solution than some existing methods in the literature.

**Table 1:** Comparison of maximum absolute errors for Example 1

$\varepsilon \downarrow N \to$	32	64	128	256	512
Our method					
$2^{ - 5}$	1.2025e−06	7.5262e−08	4.7055e−09	2.9409e−10	1.8195e−11
$2^{ - 6}$	4.2806e−06	2.6824e−07	1.6776e−08	1.0487e−09	6.5420e−11
$2^{ - 7}$	6.7000e−06	4.2429e−07	2.6515e−08	1.6583e−09	1.0359e−10
$2^{ - 8}$	3.7483e−05	2.4697e−06	1.5760e−07	9.9397e−09	6.2393e−10
$2^{ - 9}$	1.4848e−04	1.0348e−05	6.7338e−07	4.2784e−08	2.6937e−09
Results in [14]					
$2^{ - 5}$	2.3e−4	5.9e−5	1.5e−5	3.7e−6	9.2e−7
$2^{ - 6}$	3.8e−4	9.5e−4	2.4e−5	6.2e−6	1.5e−6
$2^{ - 7}$	6.4e−4	1.6e−4	4.0e−5	1.0e−5	2.5e−6
$2^{ - 8}$	1.1e−3	2.4e−4	6.9e−5	1.7e−5	4.3e−6
$2^{ - 9}$	1.9e−3	4.9e−4	1.2e−4	3.1e−5	7.6e−6

**Table 2:** Computed rate of convergence for Example 1

$\varepsilon \downarrow N \to$	32	64	128	256
$2^{ - 5}$	3.9980	3.9995	4.0000	4.0146
$2^{ - 6}$	3.9962	3.9991	3.9997	4.0027
$2^{ - 7}$	3.9810	4.0002	3.9990	4.0007
$2^{ - 8}$	3.9238	3.9700	3.9869	3.9937
$2^{ - 9}$	3.8428	3.9418	3.9763	3.9894

**Table 3:** Computed maximum absolute errors and rate of convergence for Example 2

$\varepsilon \downarrow N \to$	16	32	64	128	256
$2^{ - 7}$	1.1522e−04	7.3531e−06	4.6790e−07	2.9236e−08	1.8268e−09
	3.9699	3.9741	4.0004	4.0004	3.9998
$2^{ - 8}$	5.6563e−04	4.1555e−05	2.7502e−06	1.7562e−07	1.1074e−08
	3.7668	3.9174	3.9690	3.9872	3.9942
$2^{ - 9}$	1.8378e−03	1.5766e−04	1.1083e−05	7.2268e−07	4.5926e−08
	3.5431	3.8304	3.9388	3.9760	3.9896
$2^{ - 10}$	4.1400e−03	4.5262e−04	3.5704e−05	2.4223e−06	1.5601e−07
	3.1933	3.6641	3.8816	3.9567	3.9825

### Limitations

During the quasilinearization process, it was fixed to the first iteration. If more number of iterations were done, then the scheme can have more accurate solution than the presented results. Additional, the scheme can more illustrate the physical behaviour of the problem under consideration.

## Data Availability

No additional data is used for this research work.
